# Genetic association of miR-146a, miR-196a2, and miR-499 polymorphisms with hepatocellular carcinoma risk in an Eastern Chinese population

**DOI:** 10.3389/fonc.2026.1707963

**Published:** 2026-02-06

**Authors:** Lunjun Zhang, Qing Pang, Hongtao Wang, Tao Xu, Xiaolin Ding

**Affiliations:** 1 Department of Clinical Laboratory Science, The First Affiliated Hospital of Bengbu Medical University, Bengbu, China; 2 The Second Clinical Medical College of Anhui Medical University, Hefei, China

**Keywords:** genetic susceptibility, hepatocellular carcinoma, mir-146a, miR-196a2, miR-499, miRNA polymorphism

## Abstract

**Background:**

Hepatocellular carcinoma (HCC) ranks as the sixth most common cancer and the third leading cause of cancer-related mortality worldwide. MicroRNAs (miRNAs) are known to regulate oncogenic and tumor suppressor pathways, and single nucleotide polymorphisms (SNPs) in miRNAs may influence cancer susceptibility.

**Methods:**

We investigated the association between three miRNA SNPs—miR-146a rs2910164, miR-196a2 rs11614913, and miR-499 rs3746444—and the risk of HCC in an eastern Chinese population. A total of 353 HCC patients and 351 healthy controls were enrolled. Genotyping was performed using PCR-ligase detection reaction (PCR-LDR), and odds ratios (ORs) with 95% confidence intervals (CIs) were calculated.

**Results:**

Compared with the CC genotype, individuals carrying the CT and TT genotypes of miR-196a2 rs11614913 exhibited significantly increased risks of HCC (OR = 1.61, 95% CI: 1.10-2.37; OR: 1.66, 95% CI: 1.07-2.55). The dominant model of miR-196a2 rs11614913 also showed a significant association with HCC risk (*P* = 0.009). In contrast, carriers of the AG or GG genotype of miR-499 rs3746444 showed a reduced HCC risk (OR = 0.72, 95% CI: 0.52–0.99, *P* = 0.048). No significant association was found for miR-146a rs2910164 and HCC risk.

**Conclusion:**

Our findings suggest that miR-196a2 rs11614913 and miR-499 rs3746444 polymorphisms are significantly associated with HCC susceptibility in the eastern Chinese population and may serve as potential genetic biomarkers for early risk assessment.

## 1 Introduction

Hepatocellular carcinoma (HCC) is the sixth most common cancer and the third leading cause of cancer-related deaths worldwide, with approximately 906, 000 new cases and 830, 000 deaths reported in 2020 (1). Despite advancements in treatment options, including surgery, locoregional therapies, and systemic therapies such as sorafenib and immune checkpoint inhibitors, the prognosis for HCC patients remains poor, particularly in advanced stages. A major challenge in treating HCC lies in its late-stage diagnosis, often due to the absence of early clinical symptoms and the lack of reliable early biomarkers.

Several risk factors have been implicated in the pathogenesis of HCC, including chronic viral hepatitis, excessive alcohol consumption, exposure to aflatoxin-contaminated food, and metabolic disorders. However, only a subset of individuals exposed to these risk factors eventually develop HCC, suggesting a role for genetic predisposition (2). Recent studies have highlighted the importance of cancer biomarker discovery, particularly genetic biomarkers, in identifying individuals at high risk and enabling early, more effective interventions (3). The identification of such biomarkers remains crucial for improving early detection, treatment stratification, and overall patient outcomes.

Among genetic factors, single nucleotide polymorphisms (SNPs)—the most prevalent form of genetic variation in the human genome—have been linked to the risk of various cancers, including HCC. Genome-wide association studies (GWASs) have identified multiple SNPs associated with HCC susceptibility, further highlighting the importance of host genetic background in hepatocarcinogenesis (4–6).

In addition, microRNAs (miRNAs) are small non-coding RNAs that regulate gene expression by binding to the 3’ untranslated regions (3’ UTRs) of target mRNAs (7). SNPs in miRNA genes may influence their biogenesis or function, thereby affecting cancer development (8). Notably, three SNPs—miR-146a rs2910164, miR-196a2 rs11614913, and miR-499 rs3746444—have been implicated in the development and progression of HCC in different populations (9). However, findings have been inconsistent due to differences in ethnicity, disease stage, sample size, and study design (10, 11). A clear gap remains regarding whether these reported associations apply to the eastern Chinese Han population. Prior studies on miR-146a rs2910164, miR-196a2 rs11614913, and miR-499 rs3746444 have yielded inconsistent results, likely influenced by differences in population background and study design. Moreover, HCC etiologic exposures vary by region, suggesting that genetic effects may not be fully generalizable across cohorts. Therefore, this study provides independent evidence from an eastern Chinese cohort to refine population-specific risk estimates and evaluate the potential utility of these miRNA SNPs for risk stratification.

HCC constitutes the majority of primary liver cancers and remains a major global health burden. Despite recent advances in systemic therapies, including tyrosine kinase inhibitors, immune checkpoint inhibitors, and their combinations, which have significantly improved survival in advanced HCC, therapeutic efficacy varies widely among individuals (12–15). This variability underscores the importance of identifying molecular and genetic factors that may influence prognosis and treatment response. Genetic biomarkers such as SNPs could help stratify patients based on risk and potentially predict responsiveness to specific systemic therapies, thereby facilitating personalized treatment strategies. In this context, miRNA polymorphisms represent a promising class of biomarkers due to their roles in regulating gene expression involved in tumor progression, immune modulation, and drug resistance.

**Figure 1:**
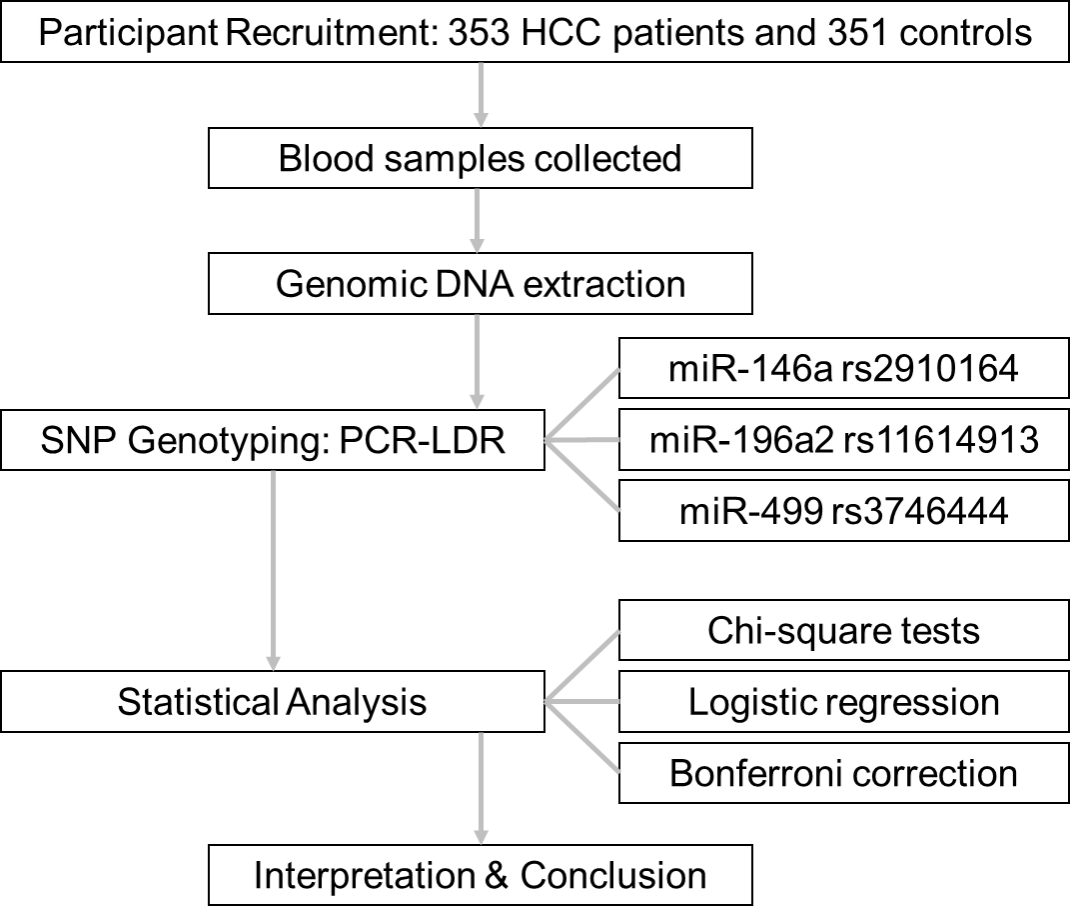
The schematic workflow.

Therefore, this study aimed to assess the association between miR-146a rs2910164, miR-196a2 rs11614913, and miR-499 rs3746444 polymorphisms and the risk of HCC in an eastern Chinese population. The schematic workflow was shown in **Figure 1**. These miRNAs were chosen based on previous studies suggesting their functional roles in cancer-related processes such as inflammation (miR-146a), cell proliferation and differentiation (miR-196a2), and apoptosis (miR-499). We further explored whether these SNPs could serve as potential genetic biomarkers for HCC susceptibility, which may aid in early screening and genetic risk stratification in clinical settings.

## 2 Materials and methods

### 2.1 Study population

This study included 353 newly diagnosed HCC patients and 351 healthy controls recruited consecutively at the First Affiliated Hospital of Bengbu Medical College between January 2019 and December 2020. HCC diagnoses were confirmed by histopathological examination of biopsy or resected tissues evaluated independently by two pathologists. Control subjects were selected from individuals undergoing routine physical examinations or outpatient visits and had no history of cancer, end-stage liver or kidney disease, endocrine disorders, or other digestive system diseases. All participants were of Chinese Han ethnicity and resided in the same geographical region. Written informed consent was obtained from all participants, and the study protocol was approved by the Ethics Committee of the First Affiliated Hospital of Bengbu Medical College (Approval No. byyfy-2018 ky33).

Inclusion criteria were: (i) newly diagnosed primary HCC during the study period; (ii) diagnosis confirmed by histopathological evaluation; (iii) Han Chinese ethnicity and residence in the same geographic region; and (iv) provision of informed consent and an adequate peripheral blood sample for genotyping.

Exclusion criteria included: (i) history of other malignancies and (ii) inability to provide complete baseline information or blood samples for analysis.

### 2.2 DNA extraction and genotyping

Peripheral venous blood samples were collected from all participants. Genomic DNA was extracted using the TIANamp Blood DNA Kit (TIANGEN, Beijing, China) following the manufacturer’s protocol. Genotyping of miR-146a rs2910164, miR-196a2 rs11614913, and miR-499 rs3746444 was performed using the polymerase chain reaction-ligase detection reaction (PCR-LDR) method by Shanghai Personalbio Technology Co., Ltd. The sequences of the PCR primers and LDR probes are listed in **Tables 1**, **2**.

**Table 1:** PCR primer sequences used in this study.

SNP	Primer name	Sequence 5′ to 3′
miR-146a rs2910164	Forward	GCCGATGTGTATCCTCAG
	Reverse	TCTCTCCAGGTCCTCAAG
miR-196a2 rs11614913	Forward	GCTGATCTGTGGCTTAGG
	Reverse	GTAGGAGTGGGAGAGGTG
miR-499 rs3746444	Forward	GGCGGCTGTTAAGACTTG
	Reverse	CACCCCTTCCCCACAAAC

**Table 2:** LDR probes sequences used for genotyping.

TagSNPs	Probes	Probes sequences (5′ to 3′)
miR-146a rs2910164	S01-TC	ATGGGTTGTGTCAGTGTCAGACCTC
	S01-TG	ctgATGGGTTGTGTCAGTGTCAGACCTG
	S01-TR	TGAAATTCAGTTCTTCAGCTGG GAT-FAM
miR-196a2 rs11614913	S02-TC	ctgaTTTGAACTCGGCAACAAGA AACTGC
	S02-TT	ctgactgTTTGAACTCGGCAACAA GAAACTGT
	S02-TR	CTGAGTTACATCAGTCGGTTT TCGTtga-FAM
miR-499 rs3746444	S03-TA	ctgactgaTGTTTAACTCCTCTC CACGTGAACA
	S03-TG	ctgactgactgTGTTTAACTCC TCTCCACGTGAACG
	S03-TR	TCACAGCAAGTCTGTGCTG CTTCCCtgactg-FAM

### 2.3 Statistical analysis

Statistical analysis was conducted using SPSS version 22.0 (SPSS, Chicago, IL, USA). Hardy-Weinberg equilibrium (HWE) in the control group was assessed using the chi-square test. Genotype and allele frequencies were compared between HCC patients and controls using chi-square tests and logistic regression models. Odds ratios (ORs) and 95% confidence intervals (CI)s were calculated for various genetic models (dominant, recessive, additive). A *P*-value < 0.05 was considered statistically significant. To account for multiple comparisons across SNPs and genetic models, Bonferroni correction was applied, adjusting the significance threshold to *P* < 0.0033 (0.05/15 tests).

## 3 Results

### 3.1 Characteristics of study subjects

**Table 3** presents the demographic and clinical characteristics of the 353 HCC patients and 351 control patients. No significant differences were observed between HCC patients and controls in terms of age, gender, and smoking status. However, alcohol consumption was significantly more prevalent among HCC patients compared with controls.

**Table 3:** The general characteristics of HCC in the studied populations.

Characteristics	Cases (n=353)	%	Controls (n=351)	%	*P* value
Age (Mean ± SD)	57.2 ± 10.20		55.34 ± 10.70		0.27
Gender					0.10
Male	243	68.84	221	62.96	
Female	110	31.16	130	37.04	
Smoking status					0.291
Never	225	63.74	237	67.52	
Ever	128	36.26	114	32.48	
Alcohol drinker					
Never	216	61.19	248	70.66	0.008
Ever	137	38.81	103	29.34	

### 3.2 Genotype distributions

The genotype distributions of miR-146a rs2910164, miR-196a2 rs11614913, and miR-499 rs3746444 in both the HCC and control groups are shown in **Table 4**. All genotype frequencies were in accordance with Hardy-Weinberg equilibrium in the control group, indicating the absence of genotyping bias.

**Table 4:** Genotype distributions of miR-146a, miR-196a2, and miR-499.

SNPs	Cases (n=353)	%	Control (n=351)	%	*P* value for HWE	
					Cases	Control
miR-146a						
GG	72	20.40	61	17.38		
GC	170	48.16	182	51.85		
CC	111	31.44	108	30.77	0.64	0.29
miR-196a2						
TT	101	28.61	89	25.36		
TC	191	54.11	173	49.29		
CC	61	17.28	89	25.36	0.071	0.79
miR-499						
GG	8	2.26	11	3.13		
AG	79	22.38	99	28.21		
AA	266	75.35	241	68.66	0.46	0.83

HWE, Hardy-Weinberg equilibrium.

### 3.3 Association between SNPs and HCC risk

**Table 5** summarizes the logistic regression analysis results between miRNA polymorphisms and HCC risk. For miR-196a2 rs11614913, both CT and TT genotypes were significantly associated with increased HCC risk compared to the CC genotype (OR = 1.61, 95% CI: 1.10-2.37; OR = 1.66, 95% CI: 1.07-2.55, respectively). In the dominant model (CT and TT vs. CC), the polymorphism was also associated with increased HCC risk (OR = 1.63, 95% CI: 1.13-2.34).

**Table 5:** Association between miR-146a, miR-196a2, and miR-499 polymorphisms and HCC risk.

Genotype	Cases, n (%)	Control n (%)	Comparison	OR (95% CI)	*P* (nominal)	*P* (Bonferroni)
miR-146a rs2910164						
CC	111 (31.44)	108 (30.77)	C vs. G	0.95 (0.77-1.18)	0.658	1.000
CG	170 (48.16)	182 (51.85)	CG vs. GG	0.79 (0.53-1.18)	0.252	1.000
GG	72 (20.40)	61 (17.38)	CC vs. GG	0.87 (0.57-1.34)	0.53	1.000
			CC vs. (CG + GG)	1.03 (0.75-1.42)	0.847	1.000
			CG + (CC vs. GG)	0.82 (0.56-1.20)	0.307	1.000
miR-196a2 rs11614913						
TT	101 (28.61)	89 (25.36)	T vs. C	1.26 (1.02-1.55)	0.033	0.495
TC	191 (54.11)	173 (49.29)	CT vs. CC	1.61 (1.10-2.37)	0.015	0.225
CC	61 (17.28)	89 (25.36)	TT vs. CC	1.66 (1.07-2.55)	0.022	0.330
			TT vs. (CT + CC)	1.18 (0.85-1.65)	0.331	1.000
			(TT + CT) vs. CC	1.63 (1.13-2.34)	0.009	0.135
miR-499 rs3746444						
AA	266 (75.35)	241 (68.66)	G vs. A	0.75 (0.56-1.00)	0.05	0.750
AG	79 (22.38)	99 (28.21)	AG vs. AA	0.72 (0.51-1.02)	0.064	0.960
GG	8 (2.26)	11 (3.13)	GG vs. AA	0.66 (0.26-1.67)	0.378	1.000
			GG vs. (AG+AA)	0.72 (0.28-1.18)	0.479	1.000
			(AG+GG) vs. AA	0.73 (0.61-0.99)	0.048	0.720

**Figure 2:**
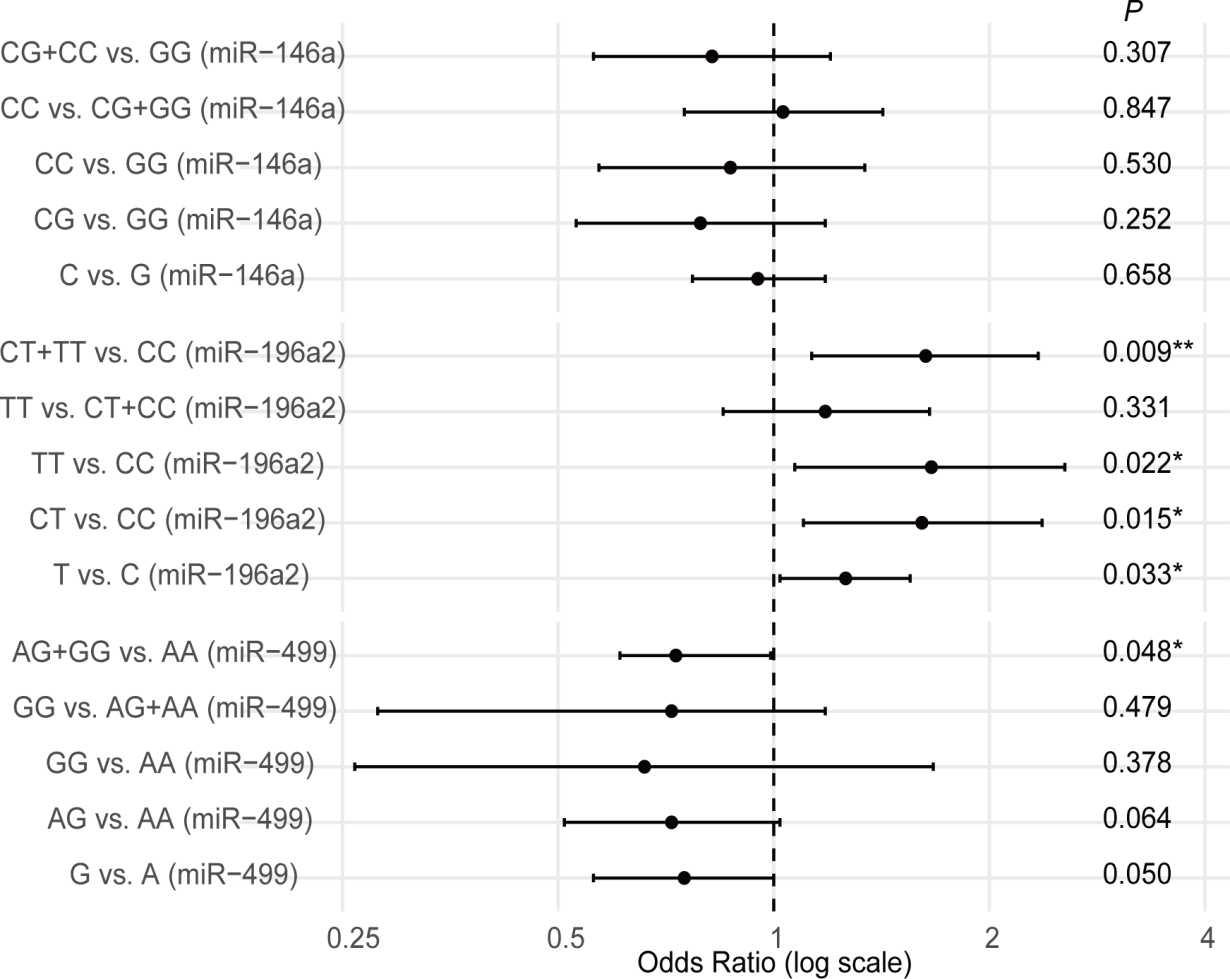
Forest plot of odds ratios (ORs) and 95% confidence intervals (CIs) for the associations between miRNA polymorphisms and hepatocellular carcinoma (HCC) risk. Each line represents one genetic comparison for miR-146a rs2910164, miR-196a2 rs11614913, or miR-499 rs3746444. The solid squares indicate the odds ratio (OR), and the horizontal lines represent the 95% confidence intervals (CI). The vertical dashed line at OR = 1.0 indicates the null hypothesis (no association). A log scale is used on the x-axis. Comparisons with *P* < 0.05 are marked in bold font in the plot.

In contrast, for miR-499 rs3746444, the dominant model (AG and GG vs. AA) was significantly associated with a decreased risk of HCC (OR = 0.73, 95% CI: 0.61-0.99, *P* = 0.048). No significant association was observed between miR-146a rs2910164 polymorphism and HCC susceptibility. These results suggest that miR-196a2 rs11614913 may increase, while miR-499 rs3746444 may reduce the risk of HCC, supporting their potential roles in disease susceptibility (**Figure 2**). After applying Bonferroni correction for multiple comparisons (n = 15 tests), none of the associations remained statistically significant at the adjusted threshold (*P* < 0.0033). Specifically, while the miR-499 rs3746444 recessive model showed a nominally significant association (*P* = 0.048), this association was no longer significant after Bonferroni correction.

## 4 Discussion

MicroRNAs (miRNAs) have emerged as pivotal regulators of gene expression and tumorigenesis, acting as either oncogenes or tumor suppressors depending on the cellular context (16). In this study, we investigated the association of three common miRNA polymorphisms—miR-146a rs2910164, miR-196a2 rs11614913, and miR-499 rs3746444—with the risk of HCC in an eastern Chinese population.

Previous functional studies support the biological relevance of these miRNAs in HCC. Our results showed that miR-499 suppresses the expression of the EST1 in HCC cells, thereby contributing to anti-tumor immune regulation (17). miR-196a may facilitate tumorigenesis by targeting oncogenes such as HOX genes, HMGA2, and Annexin A1 (18). Overexpression of miR-499 in HepG2 cells has been shown to inhibit cellular invasion and migration (19). Furthermore, dysregulation of miRNA expression has been associated with both the development and prognosis of HCC (20). Fründt et al. found that miR-146a and miR-192 levels were significantly downregulated in patients with decompensated liver cirrhosis (with and without HCC), compared to those with compensated cirrhosis (21). In addition, polymorphisms in other miRNAs such as miR-449b have been associated with decreased risk of esophageal squamous cell carcinoma in a Chinese population (22). Qi et al. also reported significant differences in miR-146a, miR-196a2, and miR-499 SNPs between HCC patients and controls (23). Together, these findings suggest that miRNA polymorphisms may serve as potential genetic biomarkers for HCC diagnosis and prognosis (24, 25).

The functional implications of these SNPs are supported by molecular evidence. The miR-146a rs2910164 polymorphism results in a G to C substitution in the precursor sequence, creating a mismatch in the miRNA stem-loop structure that may affect miRNA maturation and function. Some studies suggest that the GG genotype is associated with higher mature miR-146a expression, promoting cell proliferation in HCC (26), whereas others report increased expression with the CC genotype (27). This variation may alter the post-transcriptional regulation of target genes, although our study did not find a significant association between this SNP and HCC risk. Such discrepancies may stem from ethnic differences, as allele frequencies and linkage disequilibrium patterns vary across populations. Additionally, environmental co-factors, such as HBV infection status or dietary exposures, may modulate the penetrance of genetic variants. However, because HBV/HCV status was not systematically captured for all participants, we could not evaluate these potential interactions in the current analysis. Another possibility is that context-dependent regulation of miR-146a expression, or compensatory pathways in hepatic tissue, mitigates the impact of this SNP in certain populations. These findings suggest that genetic risk factors for HCC may not be universally applicable and highlight the need for ethnically stratified and mechanistically integrated studies.

miR-196a2 rs11614913 is located in the 3p strand of the mature miRNA sequence. The C to T transition leads to a G:C to G:U mismatch, potentially impairing miRNA processing and target recognition (28, 29). The C allele has been shown to increase mature miRNA-196a2 expression in HCC tissues (30), supporting its possible oncogenic role. Similarly, miR-499 rs3746444 is located within the stem region of the pre-miRNA and causes an A:U to G:U mismatch (31). This alteration may affect the maturation and target binding efficiency of miR-499, ultimately influencing downstream gene expression (32).

Despite extensive investigation, the reported associations between these polymorphisms and HCC risk remain inconsistent across populations. For instance, Xu et al. and Zhang et al. found that the GG genotype of miR-146a was associated with increased HCC susceptibility in Chinese individuals (26, 33), whereas other studies, including those by Yan et al., reported no significant association (34). Similar inconsistencies exist for miR-196a2 rs11614913: while Hao et al. reported a protective effect of the TT genotype (35), other studies in both Chinese and Turkish populations suggested that the C allele or CC genotype increases HCC risk (36, 37). Interestingly, Xu et al. also observed that the CC genotype correlates with higher miR-196a2 expression levels (38).

In the case of miR-499 rs3746444, the AG + GG genotypes showed a lower HCC risk than the AA genotype in a Korean population (39). However, this association was not consistently observed in studies involving Chinese cohorts (40, 41). A recent meta-analysis by Zhang et al. concluded that among the three SNPs studied, only miR-196a2 rs11614913 showed a consistent and significant association with HCC susceptibility, with the CT and TT genotypes conferring approximately 1.23-fold increased risk compared to TT homozygotes.

In the present study, we found that the CT and TT genotypes of miR-196a2 rs11614913 were significantly associated with increased HCC risk, while the AG + GG genotype of miR-499 rs3746444 was associated with a reduced risk. These findings are in line with previous functional and epidemiological studies. However, our results did not support a significant association between miR-146a rs2910164 and HCC risk in this population. The discrepancies between studies may be attributed to differences in sample size, ethnic background, study design, environmental exposures, or gene–environment interactions.

This study adds to the growing body of evidence that specific miRNA polymorphisms contribute to interindividual differences in HCC risk. The potential application of miR-196a2 and miR-499 variants as biomarkers could improve risk stratification strategies, particularly in regions with high HCC incidence. However, substantial knowledge gaps remain. For instance, the functional impact of these polymorphisms on miRNA-mRNA interactions in liver tissue remains poorly characterized. Over the next five years, we expect that the integration of high-throughput genotyping with transcriptomic and epigenomic profiling in large, multi-ethnic cohorts will help clarify these mechanisms and identify novel susceptibility loci. Furthermore, future studies combining germline SNPs with circulating miRNA levels could enhance early diagnosis and possibly inform treatment response prediction.

Beyond statistical associations, it is important to consider the biological mechanisms through which miR-146a, miR-196a2, and miR-499 polymorphisms might contribute to HCC susceptibility. For example, miR-146a plays a role in modulating the innate immune response by targeting TRAF6 and IRAK1, key adaptors in the NF-κB pathway, which is essential in inflammation-induced carcinogenesis (42). The rs2910164 polymorphism may impair the processing of pri-miR-146a, thereby influencing its regulatory capacity in hepatic inflammatory signaling (43). Similarly, miR-196a2 regulates gene clusters including HOXB and HOXC, and its rs11614913 variant, located in the mature miRNA sequence, can alter miRNA-mRNA interactions with downstream targets such as ANXA1 and HMGA2, which are implicated in cell proliferation and metastasis (44, 45). For miR-499, which modulates apoptotic and inflammatory pathways via targets like SOX6 and PDCD4, the rs3746444 SNP may influence the thermodynamic stability of the pre-miRNA hairpin and thus alter its expression levels (46). These mechanistic hypotheses highlight the potential of miRNA SNPs not only as risk markers but also as contributors to the pathophysiological heterogeneity of HCC. Future functional studies, such as CRISPR-mediated SNP editing and integrative omics profiling, are warranted to validate these regulatory effects and explore their implications in personalized HCC prevention and treatment.

Recent advances in tumor biology highlight the critical role of the tumor microenvironment (TME), including the interaction between tumor cells and cancer-associated fibroblasts (CAFs), in HCC progression. Increasing evidence suggests that miRNAs can be selectively packaged into extracellular vesicles (EVs), which mediate crosstalk between tumor and stromal cells. SNPs in miRNAs may influence not only expression but also vesicle loading, potentially altering intercellular communication. A recent study in breast cancer demonstrated how EV-miRNAs affect fibroblast activation and tumor progression (47). Although our study focused on germline SNPs, these variants might also influence the EV-mediated TME in HCC, which warrants further functional validation.

Recent progress in oncology emphasizes the role of genetic biomarkers in guiding personalized cancer treatment. Although our study focuses on miRNA polymorphisms and HCC susceptibility, these variants may also influence treatment response by affecting key pathways involved in immunity, apoptosis, and drug metabolism. As highlighted by recent reviews on emerging cancer therapies (48), integrating such genetic data may help stratify patients for targeted or immunotherapy. Future studies should explore whether miRNA SNPs can predict therapeutic outcomes, linking genetic risk with precision medicine in HCC.

Recent studies have shown that non-coding RNAs, including miRNAs and lncRNAs, may influence cancer progression through emerging pathways like ferroptosis. Although our study focused on miRNA polymorphisms, these variants could also play roles in ferroptosis regulation. Incorporating such insights into future research may help clarify the functional relevance of miRNA variants in HCC and guide novel therapeutic strategies (49).

Although our study focused on single miRNA polymorphisms, miRNAs function within complex gene regulatory networks. Recent pan-cancer studies have shown that miRNAs interact with key pathways related to DNA repair, metabolism, and cell death (50–53). Additionally, miRNAs are involved in therapy resistance, such as through the renin-angiotensin-aldosterone system in HCC and other cancers (54). These findings suggest that miRNA SNPs may have broader effects beyond individual targets. Future work combining genotyping with transcriptomic or network-based analysis could help uncover their wider roles in tumor biology and treatment response.

This study has several limitations. First, the sample size, although moderate, may limit the statistical power to detect small effect sizes, especially in subgroup analyses. Moreover, the potential for type I error due to multiple testing should be considered. While several associations showed nominal significance, none remained significant after Bonferroni correction for multiple tests. This highlights the importance of careful interpretation of these findings and suggests the need for replication in larger, independent cohorts. Second, although we observed statistically significant associations for miR-196a2 rs11614913 and miR-499 rs3746444, we did not conduct functional validation experiments such as miRNA expression profiling or target gene assays. As a result, the biological implications of these polymorphisms remain speculative and are inferred from prior literature on miRNA-mediated regulation of inflammation, apoptosis, and tumor progression. This limitation reflects practical constraints common in early-stage genetic studies, where time and resources often preclude immediate follow-up experiments (55). Nevertheless, functional validation would be critical to confirm whether these SNPs influence miRNA expression or downstream pathways in hepatocarcinogenesis. Third, our study focused exclusively on an eastern Chinese Han population, which, while epidemiologically relevant for HBV-related HCC, may limit the generalizability of our findings to other ethnic groups or geographic regions. Ethnic differences in allele frequencies, linkage disequilibrium patterns, and gene–environment interactions could influence the replicability of our results. Therefore, further studies in diverse populations are necessary to validate the observed associations and assess their global relevance. Fourth, the association observed for miR-499 rs3746444 demonstrated borderline significance, with the lower bound of the 95% confidence interval approaching 1.0. While this suggests a potential protective effect against HCC, the statistical robustness of this finding is limited. Larger, independent cohorts are necessary to confirm this association and reduce the risk of false-positive results due to limited sample size or sampling variability. Additionally, although age and gender were matched between groups, our logistic regression analyses did not account for other established HCC risk factors such as HBV/HCV infection status, alcohol intake, or metabolic comorbidities due to incomplete information in the control dataset. This may introduce residual confounding and limit the precision of the observed genetic associations. Future studies with richer clinical annotation are needed to perform multivariable adjustments and explore gene–environment interactions more rigorously. Moreover, given the strong etiologic role of chronic HBV/HCV infection in HCC, the effects of miRNA polymorphisms may differ by viral status, and formal tests of SNP–virus interactions should be prioritized in future well-phenotyped cohorts. Finally, only three SNPs were analyzed; additional miRNA loci and related regulatory elements may also contribute to HCC susceptibility. Future large-scale and integrative studies that combine genotyping with transcriptomic and functional analyses are warranted to validate and expand upon these findings.

In conclusion, our study provides evidence that miR-196a2 rs11614913 and miR-499 rs3746444 polymorphisms are significantly associated with HCC risk in the eastern Chinese population, whereas miR-146a rs2910164 does not appear to contribute to disease susceptibility. However, because the nominal associations did not remain significant after Bonferroni correction and no functional validation was performed, these findings should be considered hypothesis-generating rather than definitive. Future large-scale, multi-center studies across diverse ethnic groups are warranted to validate these associations and to further elucidate the underlying biological mechanisms.

## Data Availability

The datasets used and/or analyzed during the current study are available from the corresponding author upon reasonable request.
